# *Capparis kbangensis* (Capparaceae), a new species from central Vietnam

**DOI:** 10.3897/phytokeys.151.50477

**Published:** 2020-06-17

**Authors:** Danh Thuong Sy, Do Van Hai, Ritesh Kumar Choudhary, The Bach Tran, Hoang Mau Chu, Huu Quan Nguyen, Thi Thu Nga Nguyen, Gordon C. Tucker, Joongku Lee

**Affiliations:** 1 Faculty of Biology, TNU-University of Education, 20 Luong Ngoc Quyen, Thai Nguyen City, Vietnam TNU-University of Education Thai Nguyen City Vietnam; 2 Department of Botany, Institute of Ecology and Biological Resources, Vietnam Academy of Science and Technology, 18 Hoang Quoc Viet, Cau Giay, Hanoi, Vietnam Institute of Ecology and Biological Resources Hanoi Vietnam; 3 Biodiversity & Palaeobiology Group, Agharkar Research Institute, G.G. Agharkar Road, Pune-411-004, India Agharkar Research Institute Pune India; 4 Department of Biological Sciences, Eastern Illinois University, 600 Lincoln Avenue, Charleston, Illinois 61920-3099, USA Eastern Illinois University Charleston United States of America; 5 Department of Environment and Forest Resources, Chungnam National University, Yuseong-gu, Daejeon 34134, South Korea Chungnam National University Daejeon South Korea

**Keywords:** Capers, *Capparis versicolor*, Gia Lai Province, taxonomy

## Abstract

*Capparis kbangensis* Sy & D.V. Hai, a new species from Kbang District, Gia Lai Province, Vietnam, is described and illustrated. The new species is morphologically similar to *Capparis versicolor* but differs by several characters such as emarginate leaf apex, hairy margin of sepals, smaller fruits, and fewer seeds per fruit. Its ecology and conservation status are provided along with a taxonomic key to the closely allied species.

## Introduction

*Capparis* Tourn. ex L. is one of the largest genera of the family Capparaceae and is important due to its economic and medicinal value (Rivera et al. 2003; Jiang et al. 2007; Chedraoui et al. 2017). The genus consists of 139 species distributed in tropical and subtropical Old World to Mongolia (POWO 2019). It occupies mostly xeric habitats and is characterized by the presence of thorns and saccate outer sepals (Jacobs 1965; Hall et al. 2002, 2004). The genus is represented by the four formal sections namely *Capparis*, *Sodada*, *Monostichocalyx*, and *Busbeckea* (Jacobs 1965) (Jacobs 1965). In Vietnam, they are represented by 37 species, three subspecies and two varieties (Ho 1999; Ban and Dorofeev 2003; Sy et al. 2018). The central highlands of Vietnam possesses a rich floristic diversity and includes five provinces namely Lam Dong, Dak Lak, Dak Nong, Gia Lai and Kon Tum. Our earlier explorations in these areas have resulted in the discovery of two new *Capparis* species, namely *C. daknongensis* and *C. gialaiensis* (Sy et al. 2013, 2015). The present discovery signifies the importance of the highlands of Vietnam and demands more floristic survey and explorations in these areas.

## Material and methods

While revising the taxonomy of *Capparis* in Vietnam, a floristic exploration trip was conducted during 2011 to Kbang District of Gia Lai Province. During this trip, an interesting *Capparis* species with young flowers and ripened fruits was encountered. However, only one individual could be located at that time. During our other trips to the same area conducted during 2017–2018, another population with 11 individuals in both flowering and fruiting stage could be traced. After a thorough examination of the type materials housed at HN, K, E, P, CAL and comparison of the morphological features of the collected taxon with all *Capparis* species from South east Asia (Gagnepain 1908, 1943; Jacobs 1960, 1965; Chayamarit 1991; Raghavan 1993; Ho 1999; Ban and Dorofeev 2003; Srisanga and Chayamarit 2004; Hu 2007; Zhang and Tucker 2008; Sy et al. 2013, 2015, 2018; Fici 2016; Fici et al. 2018, 2020; Souvannakhoummane et al. 2018) led us to conclude that our species does not match perfectly with any of the existing *Capparis* and hence we describe it here as a new species.

## Taxonomy

### Capparis kbangensis

Taxon classificationPlantaeBrassicalesCapparaceae

Sy & D.V. Hai sp. nov.

2E61B775-46F7-5FF6-9FB0-DA077C496E47

urn:lsid:ipni.org:names:77209926-1

**Figure 1.**
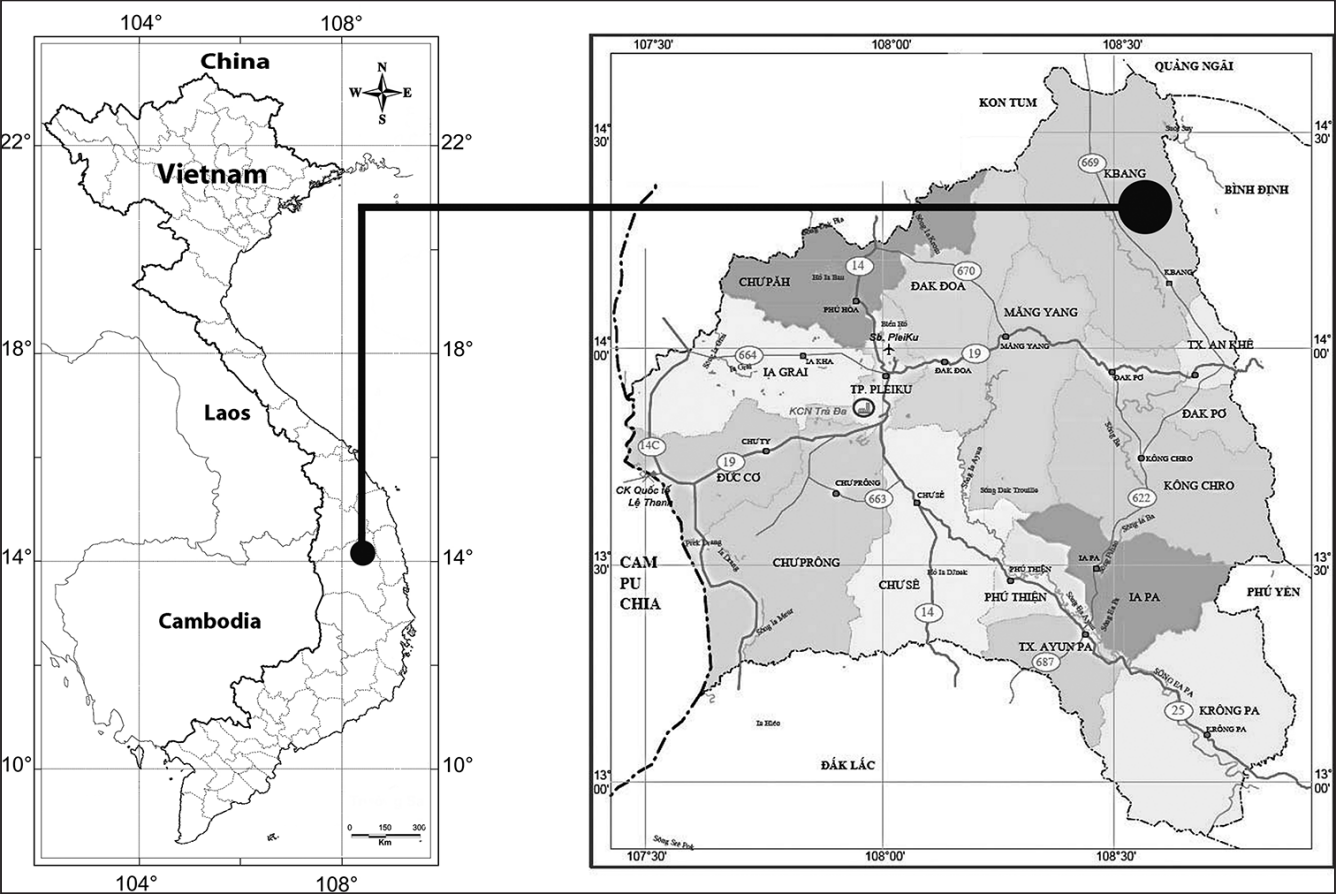
Map of Vietnam indicating type locality of *Capparis kbangensis* Sy & D.V. Hai.

**Figure 2.**
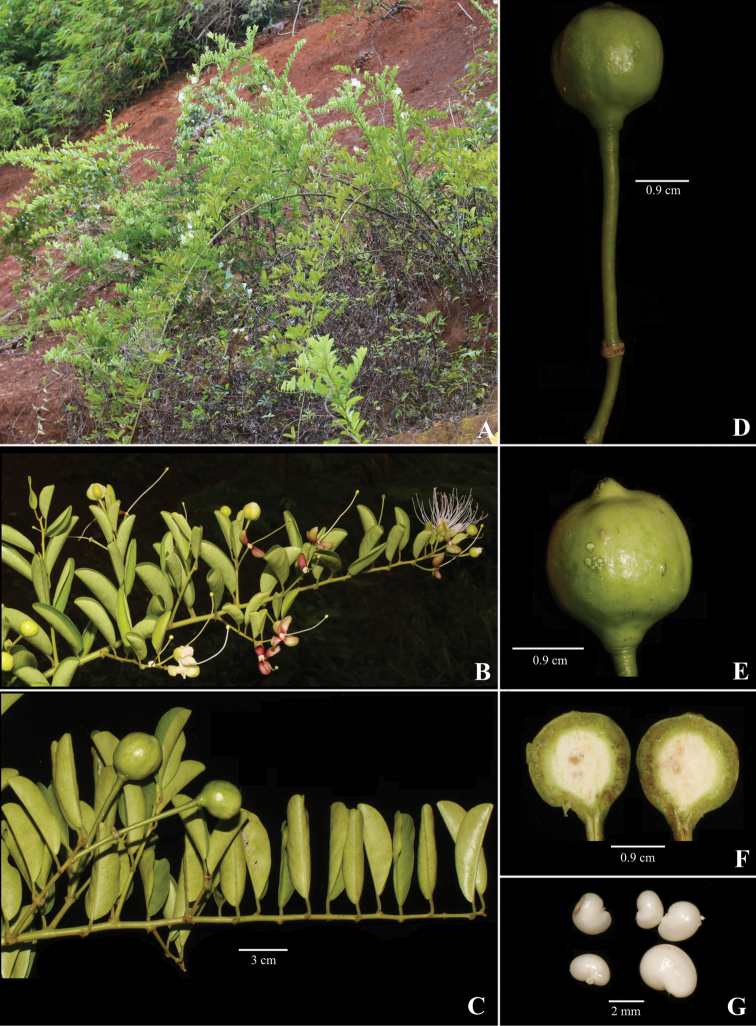
*Capparis kbangensis* Sy & D.V.Hai **A** habit **B** flowering branch **C** fruiting branch **D** fruit **E** fruit showing a distinct beak **F** cross section of the fruit **G** seeds. (Photographs by Sy & D.V. Hai).

**Figure 3.**
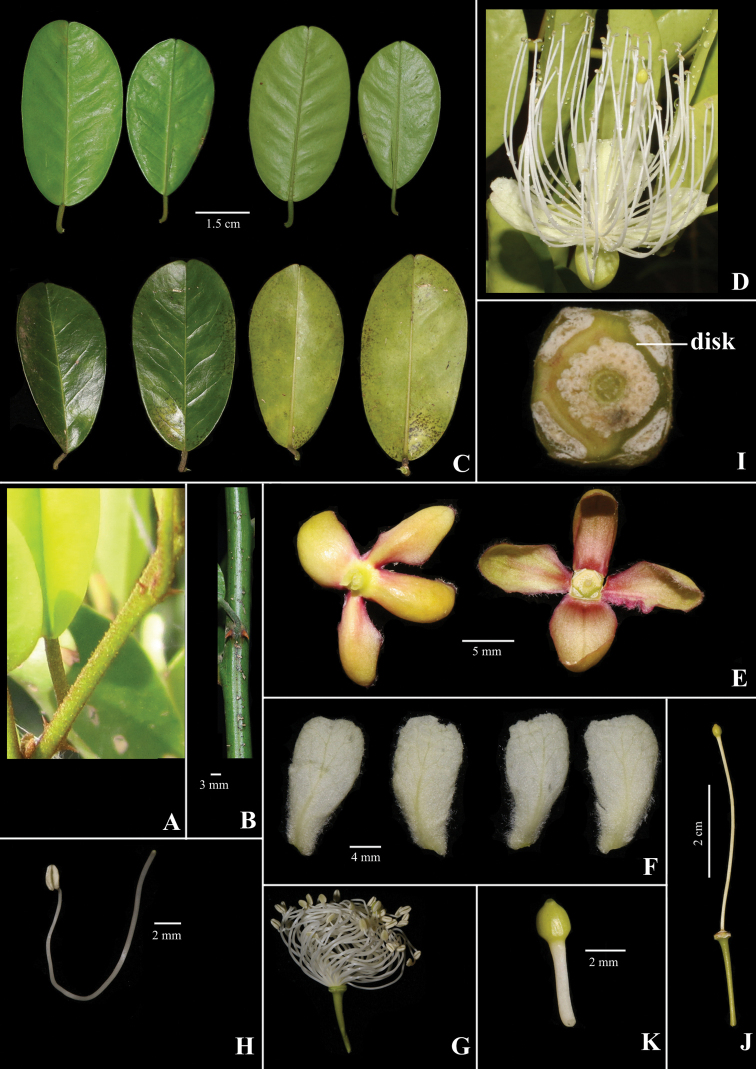
*Capparis kbangensis* Sy & D.V.Hai **A** young stem **B** mature stem showing thorns **C** leaf **D** flower **E** sepals **F** petals **G** stamens **H** stamen with filament and anther **I** disk **J** gynophore and ovary **K** ovary. (Photographs by Sy & D.V. Hai).

**Figure 4.**
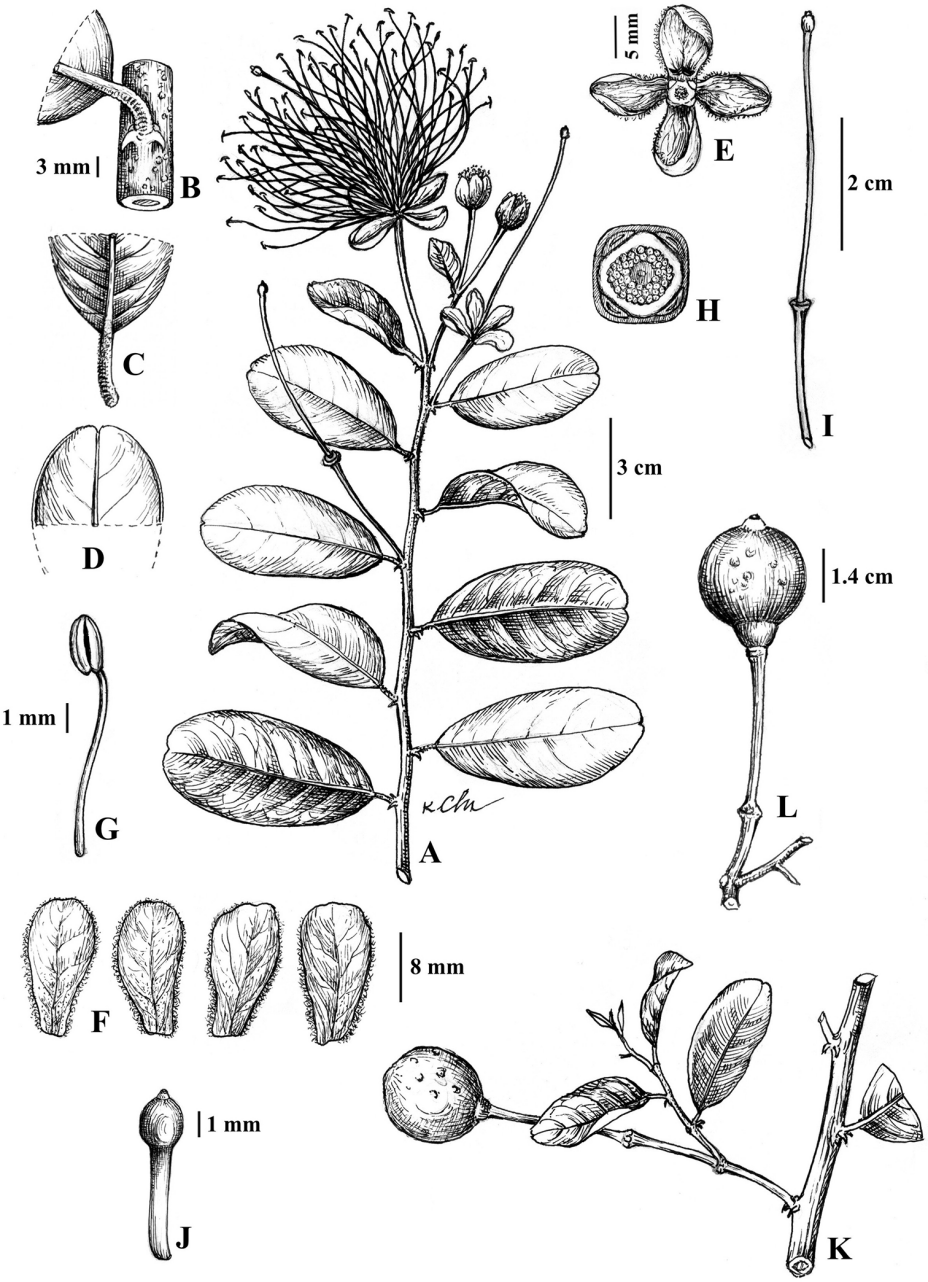
*Capparis kbangensis* Sy & D.V Hai, sp.nov (**A, C–J** drawn from holotype *Thuong*0704201801 **B** drawn from *PTV698*; **K, L** drawn from *Hai28042017*) **A** flowering branch **B** stem with thorns **C** base of leaf **D** apex of leaf **E** sepals **F** petals **G** filament and anther **H** disk **I** gynophore and ovary **J** ovary **K** fruiting branch **L** fruit (Drawn by Mrs. Le Kim Chi).

Figures 1 , 2 , 3 , 4

#### Diagnosis.

*Capparis kbangensis* is similar to *Capparis versicolor* Griff. in the number of secondary veins of the leaves, color of petals, number of stamens, the length of gynophore, but differs from it by the leaf characters (emarginate vs acute apex), hairy sepal margins (vs. glabrous), smaller fruits and number of seeds per fruit (4–5 per fruit, reniform vs. 15 per fruit, nearly polygonal).

#### Type.

Vietnam. Gia Lai Province: Kbang District, along the road, on hillocks, alt. 626 m, 14°11'44.0"N, 108°35'40.9"E, 07 April 2018, *Sy Danh Thuong, Do Van Hai, Thuong 0704201801* (holotype HN!; isotype, IBSC!).

#### Description.

Scandent shrubs, up to 5 m high. Innovations with densely brown hairs, glabrous when mature. Thorns ca. 3 mm long, reddish brown, recurved. Petiole 6–8 mm long, hairy; blade elliptic to obovate, 4.5–6 × 2.8–3.2 cm, glabrous, young leaves yellowish green, dark green when older; midvein abaxially raised, adaxially flat; secondary veins 6–7 on each side of midvein, abaxially not obvious; base round or cuneate; apex emarginate. Inflorescence corymbs simple, terminal, with the lower flowers axillary, or few flowered in lateral racemes; pedicels 1.5–1.7 cm long, glabrous. Flower buds globose, 5–6 mm. Sepals 0.9–1 × 0.4–0.5 cm long, outer pairs hairy along margins, inner pairs hairy inside and along the margins; sepals of outer whorl boat-shaped and inner whorl obovate. Petals white, later turns to light pink, obovate, lower pairs 1.6–1.7 × 0.7–0.9 cm, upper pairs 1.7–1.8 × 0.9–1 cm, both surfaces pubescent, especially at the base. Stamens 57–60; filaments 3–3.5 cm long, glabrous, white; anthers ca. 2 mm long. Disk nearly parallelogram shaped. Gynophore 3.8–4.2 cm long, glabrous. Ovary elliptic, 0.2 × 0.1 cm, top with beak, yellowish green, glabrous, placentas 4. Fruits globose, 1.8–1.9 cm in diam, black when mature, surface with some knobs, beaked apex. Seeds 4–5 per fruit, reniform, 4–5 × 2–3 mm.

#### Other specimens examined.

Vietnam. Gia Lai Province, Kbang District, near the edge of forest, alt. 630 m, 14°12'16.0"N, 108°36'18.5"E, 11 March 2011, *T.T. Bach, D.V. Hai, B.H. Quang, H.M. Tam, S.D. Thuong, PTV 698* (HN!). VIETNAM. Gia Lai Province, Kbang District, along the road, on the small hillocks, alt. 626 m, 14°11'44.0"N, 108°35'40.9"E, 28 April 2017, *D.V. Hai, Hai28042017* (HN!).

#### Phenology.

Fls. March to April; Frts: April to August.

#### Distribution and ecology.

*Capparis kbangensis* is currently known from Kbang District, Gia Lai Province. It was found growing on the basaltic soils of hillocks along the roads or near the edge of forests, at an elevation of around 626–630 m. *Capparis micracantha* DC., *Saccharum spontaneum* L., *Chromolaena odorata* (L.) R.M. King & H. Rob., *Erycibe* sp., *Callicarpa albida* Bl., *Bidens pilosa* L. were found associated with the new species.

#### Etymology.

The new species derives its name from the type locality Kbang District of the Gia Lai Province in Vietnam. In Vietnamese, it is known as Cáp kbang.

#### Conservation status.

During the last 10 years of our survey in Vietnam forests, we could observe only 12 individuals of *Capparis kbangensis* growing along the road or near the edge of forests. These habitats are frequently affected by the anthropogenic activities. Locating this species in the nearby areas proved a futile exercise. Therefore, *Capparis kbangensis* is assessed as Critically Endangered (CR) or Data deficient (DD) based on the IUCN Red List Categories (IUCN 2017). Further inventories are needed to find additional populations in Vietnam.

#### Note.

*Capparis kbangensis* is morphologically similar to *Capparis versicolor*. However, it also exhibits some similarities with *C. monantha*, *C. siamensis*, and *C. flavicans*. A comparison of the morphological characters of these species belonging to section Monostichocalyx is summarized in Table 1.

**Table 1.** Morphological comparison of *Capparis kbangensis* with others species of section Monostichocalyx.

Characters	*Capparis kbangensis*	*Capparis versicolor*	*Capparis monantha*	*Capparis siamensis*	*Capparis flavicans*
**Shape of leaf**	elliptic, nearly obovate or obovate	elliptic, oblong	ovate, elliptic,	ovate	obovate, elliptic, rhomboid
**Size of leaf (cm)**	4.5–6 × 2.8–3.2	3.5–8 × 1.5–3.5	4–7 × 2–4	5–10.5 × 3–4.5	1.2–3 × 1–1.7
**Leaf surface**	glabrous	glabrous	hairy when young, soon glabrous	hair on the midvein and secondary vein	densely hairy when young, soon glabrous
**Leaf base**	round or cuneate	cuneate	obtuse, acute	cordate, obtuse, round	obtuse, cuneate, acute
**Leaf apex**	emarginate	acute, obtuse, V-shape or acuminate	acute-acuminate, mucronate	acute or acuminate	round, obtuse, notched, mucronate
**Secondary veins (in pairs)**	6–7	6–9	2–4	4–7	3–5
**Inflorescence**	simple corymb	simple corymb	solitary	solitary	solitary
**Length of pedicels of flowers (in cm)**	1.5–1.7, glabrous	1.5–5, glabrous	0.5–1.5, glabrous	1–1.3, hairy	1–3, hairy
**Shape of sepal**	outer pairs boat-shape. inner pairs obovate	outer pairs boat-shape or nearly orbicular. inner pairs elliptic	both outer and inner pairs obovate	both outer and inner pairs elliptic or boat-shaped	outer pairs boat-shape, inner pairs ovate or obovate
**Size of sepal (in cm)**	0.9–1 × 0.4–0.5	0.9–1.1 × 0.8–1	1.4–1.8 × 0.6–0.8	0.8–1 × 0.3–0.5	0.6–0.8 × 0.4–0.5
**Sepal surface**	outer pairs only hairy along the margin. inner pairs hairy inside and along the margins	outer pair and inner pair glabrous	outer pair and inner pair only hairy outside	outer pairs hairy. inner pairs hairy outside	inner pair more hairy than outside
**Shape of petal**	obovate	nearly orbicular, obovate	obovate	obovate, sometimes spathulate	obovate
**Size of petal (cm)**	inner pairs 1.6–1.7 × 0.7–0.9; outer pairs 1.7–1.8 × 0.9–1; both surface hairy, especially at the base	1.2–1.7 × 0.7–1.4, glabrous or inside hairy near base	2.5–2.8 × 0.8, hairy outside	2–2.5 × 0.5–0.8, hairy outside	0.8–0.9 × 0.4, densely hairy outside
**Petal color**	white, turns light pink on maturity	white, purple, pink	white with yellow	green, white, upper pairs with a deep yellow blotch, fading red	yellow, upper pairs with golden yellow blotch, fading brown
**Stamens**	57–60	50–70	more than 46	36–46	6–12
**Ovary**	elliptic, 0.2 × 0.1 cm, top with knob, glabrous	elliptic, 0.2 cm long, glabrous	elongate, 0.5 × 0.1 cm, long beak, hairy	ovate, 0.3–0.5 × 0.25 cm; beaked, densely yellowish hairy	ovate, obovate, densely hairy
**Gynophore**	3.8–4.2 cm, glabrous	3–5 cm, glabrous	2.1–2.4 cm, hairy	2–2.5 cm, hairy	1.2–1.7 cm, hairy
**Fruits**	globose, diam. 1.8–1.9 cm, surface glabrous with some protuberances, beaked	globose, diam. 3–5 cm, surface glabrous or scabrous, sometimes with a few small irregular protuberances, beaked	unknown	nearly globose, elliptic or ovate; 3.5–5.5 × 2.5–3 cm, with 8 longitudinal rows of small protuberances, sometimes glabrous	subglobose, elliptic, 2.5–4 × 2–3.5 cm, surface with densely small protuberances
**Seeds**	4–5 per fruit, reniform	15 per fruit, nearly polygonal	unknown	0.7–1 × 0.5–0.6 cm	2–8 per fruit, elliptic, reniform
**Distribution**	Vietnam	China, India, Malaysia, Myanmar, Thailand, Vietnam	Thailand	Cambodia, Vietnam, Thailand	Cambodia, India, Malaysia, Myanmar, Thailand, Vietnam
**Habitat**	on the basaltic soils of the hillocks along the roads or near the edge of forests	on slightly dry areas, sandy areas, scattered forests or among shrubs	on limestone hills	on mixed deciduous forest, bamboo forest, open dry jungle, edge of evergreen forest	dry scrub, deforested land, evergreen jungle, dry *Dipterocarp* forest on poor sandy or rocky soil
**Elevation (m asl)**	626–630	100–1000	100	50–1200	40–350
**Phenology**	Fls: March-April	Fls: April-July	Fls: February-August	Fls: December-April	Fls: December-April
	Frts: April-August	Frts: July-April	Frts: unknown	Frts: May-November	Frts: May-November

### Key to the *Capparis kbangensis* and allied species

**Table:** 

1	Stamens 6–12	***C. flavicans***
–	Stamens more than 30	**2**
2	Leaf hairy. Ovary elongate or ovate, hairy	**3**
–	Leaf glabrous. Ovary elliptic, glabrous	**4**
3	Sepal obovate. Petals yellowish-white. Ovary elongated	***C. monantha***
–	Sepal boat-shaped. Petal greenish-white. Ovary ovate	***C. siamensis***
4	Apex of the leaf acute, obtuse, V-shape or acuminate. Inner pairs of sepals elliptic, glabrous. Fruits globose, diam. 3–5 cm. Seeds 15 per fruit, nearly polygonal	***C. versicolor***
–	Apex of the leaf emarginate. Inner pairs of sepals obovate, hairy. Fruits globose, diam. 1.8–1.9 cm. Seeds 4–5 per fruit, reniform	***C. kbangensis***


## Supplementary Material

XML Treatment for Capparis kbangensis
